# RHYTHM: An Open Source Imaging Toolkit for Cardiac Panoramic Optical Mapping

**DOI:** 10.1038/s41598-018-21333-w

**Published:** 2018-02-13

**Authors:** Christopher Gloschat, Kedar Aras, Shubham Gupta, N. Rokhaya Faye, Hanyu Zhang, Roman A. Syunyaev, Roman A. Pryamonosov, Jack Rogers, Matthew W. Kay, Igor R. Efimov

**Affiliations:** 1 The George Washington University, Department of Biomedical Engineering, Washington, 20052 USA; 2 The University of Alabama at Birmingham, Department of Biomedical Engineering, Birmingham, 35294 USA; 3 Moscow Institute of Physics and Technology, Dolgoprudny, 141701 Russia; 4 Institute of Numerical Mathematics of the Russian Academy of Sciences, Moscow, 119991 Russia

## Abstract

Fluorescence optical imaging techniques have revolutionized the field of cardiac electrophysiology and advanced our understanding of complex electrical activities such as arrhythmias. However, traditional monocular optical mapping systems, despite having high spatial resolution, are restricted to a two-dimensional (2D) field of view. Consequently, tracking complex three-dimensional (3D) electrical waves such as during ventricular fibrillation is challenging as the waves rapidly move in and out of the field of view. This problem has been solved by panoramic imaging which uses multiple cameras to measure the electrical activity from the entire epicardial surface. However, the diverse engineering skill set and substantial resource cost required to design and implement this solution have made it largely inaccessible to the biomedical research community at large. To address this barrier to entry, we present an open source toolkit for building panoramic optical mapping systems which includes the 3D printing of perfusion and imaging hardware, as well as software for data processing and analysis. In this paper, we describe the toolkit and demonstrate it on different mammalian hearts: mouse, rat, and rabbit.

## Introduction

Cardiac mapping is a powerful technique to understand the spread of electrical activity during normal cardiac rhythm as well as during abnormal electrical behavior such as initiation and maintenance of arrhythmias. Traditionally, electrical mapping with surface electrodes has been used for cardiac mapping. This method, however, is constrained by the number of electrodes that can be placed on the cardiac surface (low spatial resolution) and maintenance of good electrode-tissue contact (low quality of signals)^1^. Moreover, the interpretation of electrical signals during complex arrhythmias and antiarrhythmic therapies is challenging and ambiguous due to noise, far field sensing, and the method’s susceptibility to stimulation artifacts (e.g., defibrillation shocks)^2^. Optical imaging with fluorescent probes (e.g., calcium and voltage sensitive dyes) overcomes these problems, facilitating high resolution cardiac mapping without direct tissue contact^3,4^. Consequently, optical mapping techniques have revolutionized cardiac electrophysiology research, advancing our understanding of cardiac electrical activity, calcium handling, signaling, and metabolism^5–8^.

While many electrophysiological studies can be carried out using a monocular setup, there are some that necessitate a whole surface approach to data acquisition. Of particular relevance are ventricular and atrial arrhythmias. It has been observed that meandering reentrant rotors and/or dynamic focal sources of activity perpetuate arrhythmias^9,10^. Assessment of these rotors and sources of activity would be limited to those on the camera facing epicardial surface, that do not meander outside the field of view. Panoramic optical mapping overcomes these limitations by first visualizing the entirety of the heart surface and then eliminating discontinuities in the data by projecting it onto a representative, organ specific, anatomical geometry.

Several laboratories have developed different approaches to panoramic imaging as imaging technology continued to improve. Lin *et al*. used two mirrors to project multiple views onto a single CCD camera sensor^11^. However, the method was constrained by the absence of geometric information and relatively low quality of recordings. Bray *et al*. introduced the ability to reconstruct realistic epicardial geometry to visualize functional data from small rabbit hearts^12^. Kay *et al*. extended the technique to image functional data from larger swine hearts by four CCD cameras^13^. Efimov laboratory has progressively developed several generations of panoramic imaging systems and introduced computational 2D to 3D translation methods to quantify and characterize arrhythmia behavior^14^.

Over the last decade, panoramic imaging has been used to effectively investigate wavefront and rotor dynamics during ventricular tachycardia and fibrillation^15–18^, approaches to low-voltage electrotherapy^19,20^, and drug-induced arrhythmia maintenance^21^. Additionally, an increase in the investigation of atrial fibrillation (AF) and ventricular fibrillation (VF) initiation and maintenance using transgenic mouse models^22–24^ has created the need for panoramic setups that are easily scalable to accommodate small rodent hearts, such as mouse and rat. In spite of these accomplishments and a growing unmet need, the technical expertise required to design and implement a panoramic imaging system has made it largely inaccessible. We present here an open source toolkit (https://github.com/optocardiography) for building panoramic optical mapping systems capable of imaging small mammal hearts; this includes instructions for the 3D printing of experimental components and software for data acquisition, processing and analysis. Our aim is to provide a solution a cardiovascular scientist, without specialized computer science and engineering training, can easily access and implement. The toolkit will be released under the MIT open source software license.

## Results

### System Requirements

The software requirements for running panoramic imaging software are LabVIEW (version 14 or above), MATLAB (2016b or above), and MATLAB compatible C compiler (XCode 8.0 for Mac OS, MinGW 5.3/Visual Studio 2010 or above for Microsoft Windows or later, gcc-4.9 for Linux). The recommended hardware requirements include Intel CPU Core i5-2500K 3.3 GHz or comparable processor, AMD Radeon 2 GB or comparable graphics card, 16 GB or above memory (RAM), and 1 TB or above of disk space.

### Software Architecture

**Figure 1:**
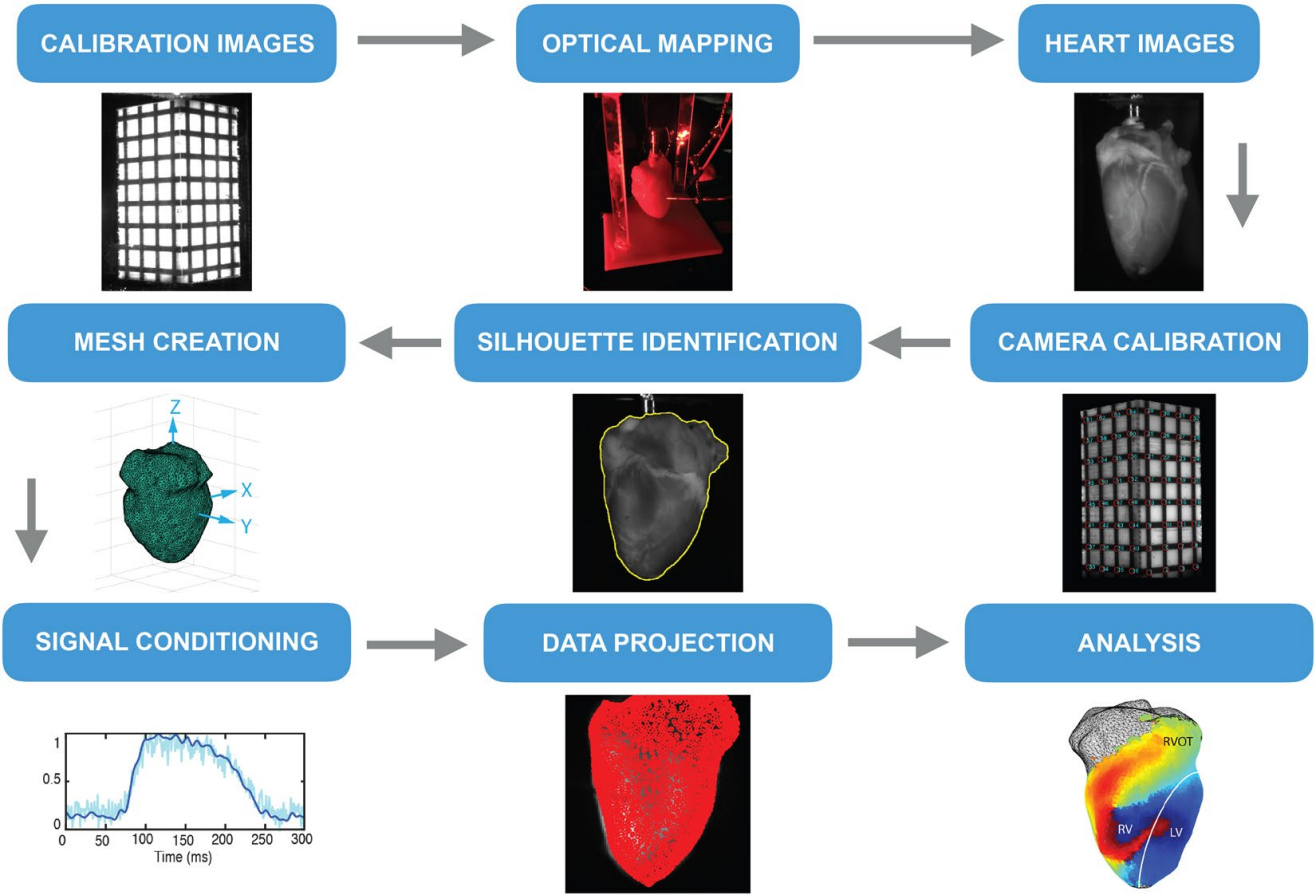
RHYTHM panoramic imaging workflow. Producing panoramic data requires a detailed oriented, step-by-step process. RHYTHM provides a concise and intuitive set of tools for accomplishing this.

Figure 1 depicts the workflow for the RHYTHM toolkit that includes an open source software module for geometry reconstruction, optical signal processing, and visualization. Software for rotating the heart following the optical mapping study is done using Labview and has been tested on Labview version 14.0. Graphical user interfaces (GUI) were created in Matlab for (1) camera calibration, (2) surface reconstruction, and (3) projection and analysis of the optical data. The layout of each GUI guides the user through the process of generating the desired output. Semi-automated routines have been implemented, where feasible, to make data processing faster and less labor intensive. A manual providing a detailed description of each GUI is included in the data supplement. Silhouette images collected from rotating the heart are saved as *.tiff files. Once camera calibration has occurred all files are saved in MATLAB’s proprietary *.mat file format. To provide readers with a 3D printing foundation, the toolkit also includes design specifications for building a panoramic optical imaging system.

Camera Calibration. We established the camera calibrations before conducting the experiments. The low-cost geometry camera (UI-322XCP-M, IDS Imaging Development Systems, Obersulm, Germany) used the same window as the optical camera A. To maintain the integrity of these cameras’ calibrations, the geometry camera was first calibrated from a bracketed position and then moved to the side. Optical mapping camera A was then calibrated and left in place for the duration of the experiment. During calibration, white LEDs (Joby Gorillatorch, DayMen US Inc, Petaluma, CA) were positioned at the illumination windows to increase the contrast between the cuboid and the calibration grid. These were later replaced by the red LEDs for the optical mapping study. Upon terminating the experiment, the geometry camera was returned to the bracketed position for collection of the silhouette images (see Geometric Reconstruction).

The method for establishing pixel-to-geometry correspondence has already been described in detail^12–14^. Briefly, we first solved for the unknown parameters of a perspective camera model^25^. This was done by first imaging an object of known dimensions with easily identifiable landmarks whose positions on the object’s surface were also known. For the rabbit heart we used a rectangular cuboid with 1 × 1 × 2 (L × W × H) inch dimensions. Smaller cuboids were used for mouse and rat hearts. A grid was placed on the outward facing surfaces providing each camera with sixty-four visible intersections, when the cuboid was placed such that two faces were visible from each camera.

**Figure 2:**
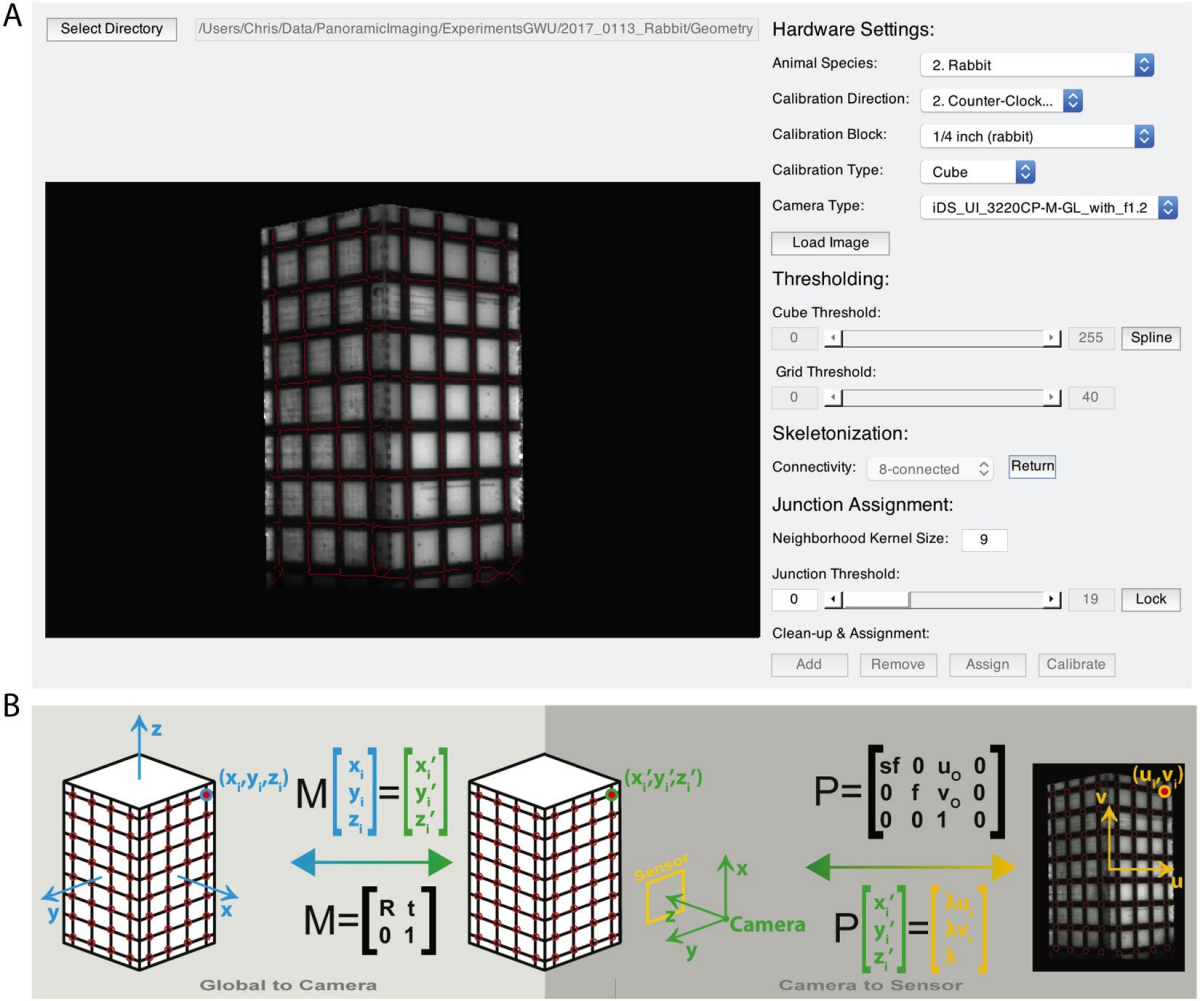
Calibration of geometry and optical cameras. (**A**) A MATLAB graphical user interface was used to semi-automate the calibration of each camera needed to facilitate the projection of optical data onto the geometric surface. Detailed explanations and code are included in the toolkit. (**B**) The cuboid is used to create a global coordinate system whose origin is at the cuboid’s center. All grid junctions on the cuboid surface have known coordinates in the global coordinate system. Identification of these points in a 2D image provides the necessary number of known values to solve for the unknown components of the global-to-camera (i.e., transformation) and the camera-to-sensor (i.e., perspective projection) matrices.

We created a graphical user interface (GUI) to ease the process of establishing the calibration (Fig. 2A). In the ideal scenario (i.e., all intersections visible), an automated algorithm identified and appropriately labeled all intersections based on a user created mask of the cube and its grid. Tools are provided that allow the user to correct poorly identified junctions prior to labeling. In the non-ideal situation (i.e., not all intersections are visible), the user is queried for the number of a visible junction in the top-left and bottom-right corner, and all other visible junctions are then automatically labeled. Detailed descriptions of the algorithms used to accomplish this are included in the toolkit. A Levenberg-Marquardt nonlinear optimization algorithm receives the 2D junction locations for each camera and the corresponding 3D intersection values and iteratively solves for the unknown extrinsic and intrinsic camera parameters needed to create transformation matrices to connect the two data sets.

The extrinsic camera parameters include the camera position (*t*) and rotation (*R*) with regards to the global coordinate system created by the cuboid. These translation and rotation values comprise the transformation matrix (*M*) as seen in Fig. 2B. The transformation matrix from the camera coordinate system to the imaging plane, also known as the perspective transformation (*P*), utilizes the intrinsic parameters focal length (*f*), aspect ratio (*s*), and the image center values (*u*_*o*_, *v*_*o*_). The final component of the transformation corrects for lens distortion. Our setup, as described above, experiences little to no lens distortion, however, this component of the algorithm is still included in the calibration GUI and is described in detail in previous work. The optimization algorithm is run for all four optical cameras and the geometric camera, providing each with a unique set of transformation matrices that will later be used for projection of data onto the reconstructed geometry.

### Surface Generation

**Figure 3:**
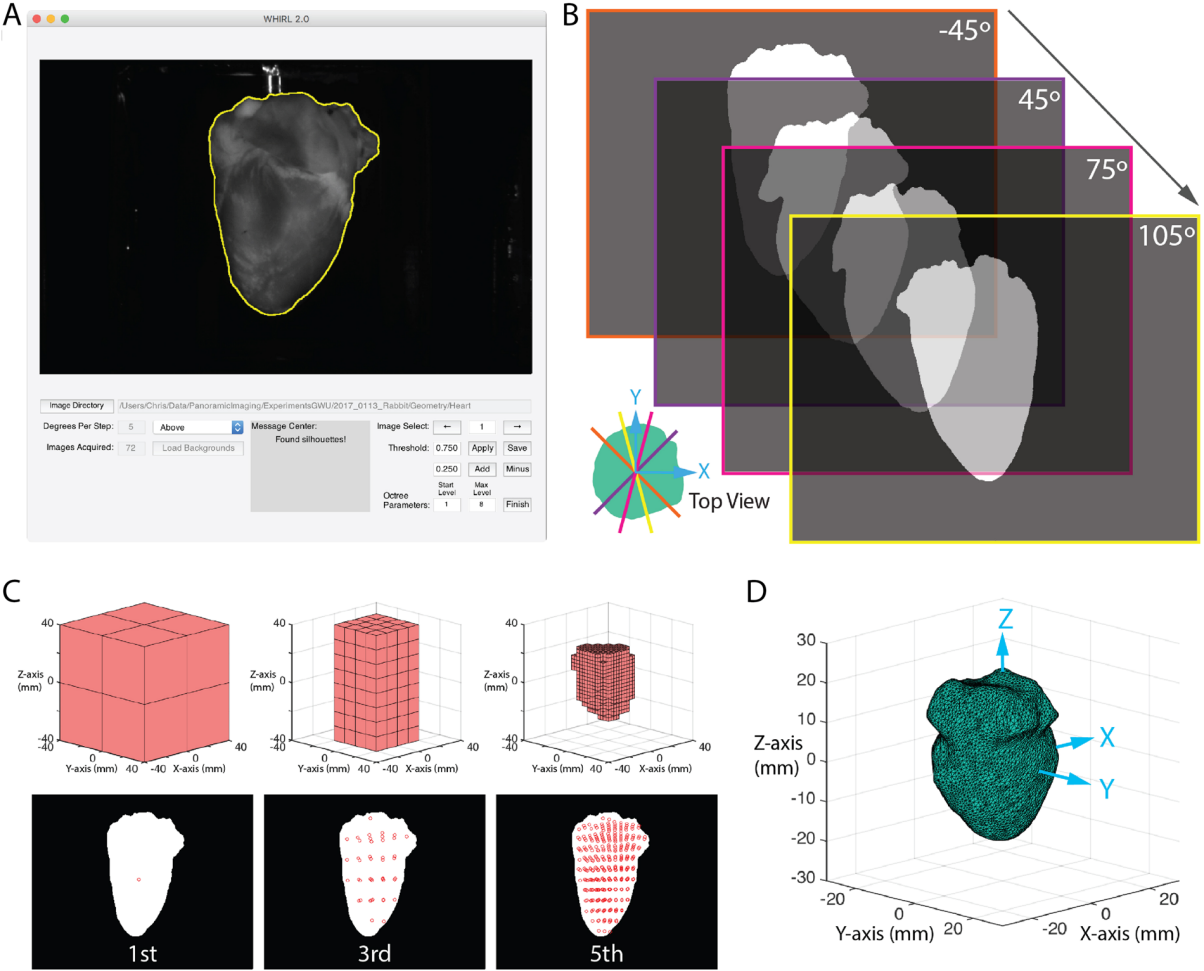
Geometric reconstruction. (**A**) A MATLAB graphical user interface was developed to facilitate accurate identification of the heart silhouettes needed for geometric reconstruction. A detailed description and the code are included in the toolkit. (**B**) The heart was rotated, and binary silhouette images were collected every 5 degrees. (**C**) Using the occluding contours method paired with an octree algorithm a series of voxels are iteratively broken down to identify the heart volume. (**D**) The surface mesh is generated by MATLAB from this volume and smoothed by external C function.

Upon completion of the optical mapping studies, we removed Camera A and returned the geometry camera to its bracketed position. The LEDs nearest the geometry camera were replaced with white LEDs and a backdrop was placed behind the heart to improve contrast. Using a custom-built LabVIEW program, we rotated the motorized rotational stage, to which the cannula was attached, 360 degrees, collecting an image of the heart every five degrees. We then uploaded these images to our custom written MATLAB GUI (Fig. 3A) that facilitates the identification of the heart silhouette in each image using a combination of thresholding and polyline tools. Heart pixels are represented as ones and background pixels as zeros (Fig. 3B).

We then used the occluding contour method^26,27^, in conjunction with an octree algorithm, to efficiently identify the volume represented by the silhouettes in a similar fashion as previously described work^13^. Briefly, an octree is used to deconstruct a volume, slightly larger than that of the heart and centered at the world coordinate system origin, into eight constituent voxels stacked on top of one another as seen in the first iteration of Fig. 3C. The vertices of each voxel are projected onto each of the silhouettes in succession by rotating the volume five degrees between silhouettes. Voxels whose vertices are either completely inside the heart (i.e. all assigned a one) or outside the heart (i.e. all assigned a zero) remain unchanged. Voxels who lie along the border (i.e. whose pixels are a mix of ones and zeros) are broken down using the octree. The process is repeated until the desired resolution is reached.

Once this was accomplished, the resulting volume was used to generate a triangulated surface mesh in MATLAB in combination with a smoothing algorithm^28^ implemented in C^29^. The result is a triangular mesh (Fig. 3D) representative of the epicardium with *N*_*cells*_ cells (i.e. triangles) and *N*_*verts*_ vertices.

### Projection and Visualization

**Figure 4:**
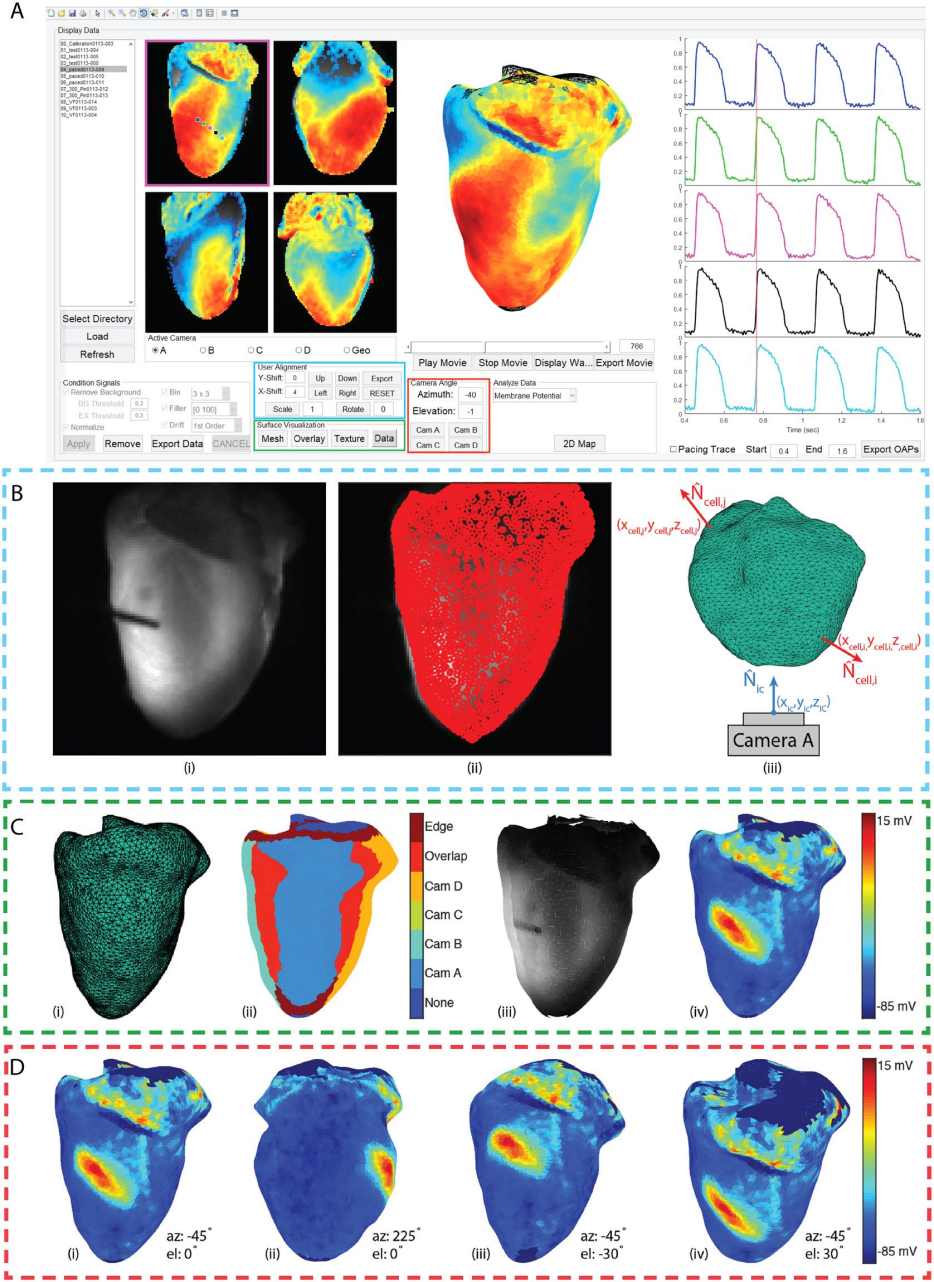
Data processing and projection (**A**) A final MATLAB graphical user interface was created to facilitate the processing of optical data and its projection onto the representative geometry. (**B**) Projection is accomplished by projecting the face centroids of the triangulated mesh onto the optical mapping cameras. Tools are provided to correct minor inaccuracies in projection. Camera facing centroids are first identified as those whose normals (*N*_*cell*_) creates an angle greater than 90° with the camera normal (*N*_*ic*_). (**C**) The surface can then be visualized as a triangulated mesh (i), a map of the camera assignments made to each centroid (ii), texture (iii), and membrane potential (iv). (**D**) Once correspondence has been established the geometry will appear with membrane potential mapped to the surface. Tools are provided to rotate the heart using click-and-drag, to the view from each camera, and to specific viewing angles using azimuth and elevation angles.

Projection of the data onto the heart surface by texture mapping^30^ and subsequent analysis was facilitated with a final GUI (Fig. 4A) that builds on the foundation of our previously published work on the analysis of single camera optical mapping signals^31^. We first load in a complete data set and remove background pixels lacking physiological data. We then normalized the optical action potentials, performed spatial filtering by binning with a 3 × 3 box shaped averaging kernel, applied a 100th order FIR temporal filter with a band pass of 0–100 Hz, and performed 1st order drift removal as necessary. We have also included a polyline tool to facilitate the removal of undesired regions (e.g. the atria when the focus of the study is the ventricles). The final step before projection is the creation of an image mask. Since surface curvature at the edge of each image decreases the signal-to-noise (SNR) ratio and introduces artifacts^32^, a weighted gradient was created for each mask with center pixels being assigned values of 1 and the outermost edge pixels being assigned values of 0.5.

Once conditioned and masked, the centroids of the triangles on the mesh are projected onto each optical mapping camera using the transformation matrices derived from previously described camera calibrations (Fig. 4B(i),B(ii)). Assignments are made using the methodology of Kay *et al*.^13^. Briefly, for each camera the angle between the camera view (*N*_*ic*_) and each of the mesh cells (*N*_*cell*_) is calculated (Fig. 4B(iii)). Minor inaccuracies in projection can be adjusted using the User Alignment tools in the GUI. All centroids with angles less than 90° are excluded from assignment at that camera. Centroids with angles greater than 115° are assigned as the signal from the closest pixel of that particular camera (e.g. Cam A, B, C, or D). Centroids assigned to multiple cameras are considered overlap pixels. The signals at these locations are calculated as a weighted average of the two assignments using the weighted gradient mask described earlier. Finally, the outermost edge cells, with angles 90° and 115°, are assigned as the edge identifier and signals are calculated as an edge weighted average.

Once projected, the geometry surface visualization tools (Fig. 4A) can be used to view the 3D surface as a triangulated untextured mesh (Fig. 4C(i)), an assignment map (Fig. 4C(ii)), an anatomical texture map (Fig. 4C(iii)), or a texture map of raw fluorescence (Fig. 4C(iv)). The axes where the 3D surface is visualized are automatically set to allow the user to rotate the model. Azimuth and elevation angles are automatically updated in the Camera Angle tool bar as the user rotates the heart or can be entered manually (Fig. 4D(i–iv)). Additionally, buttons have been provided to take the user directly to the view from each of the optical mapping cameras.

### Software Validation

**Figure 5:**
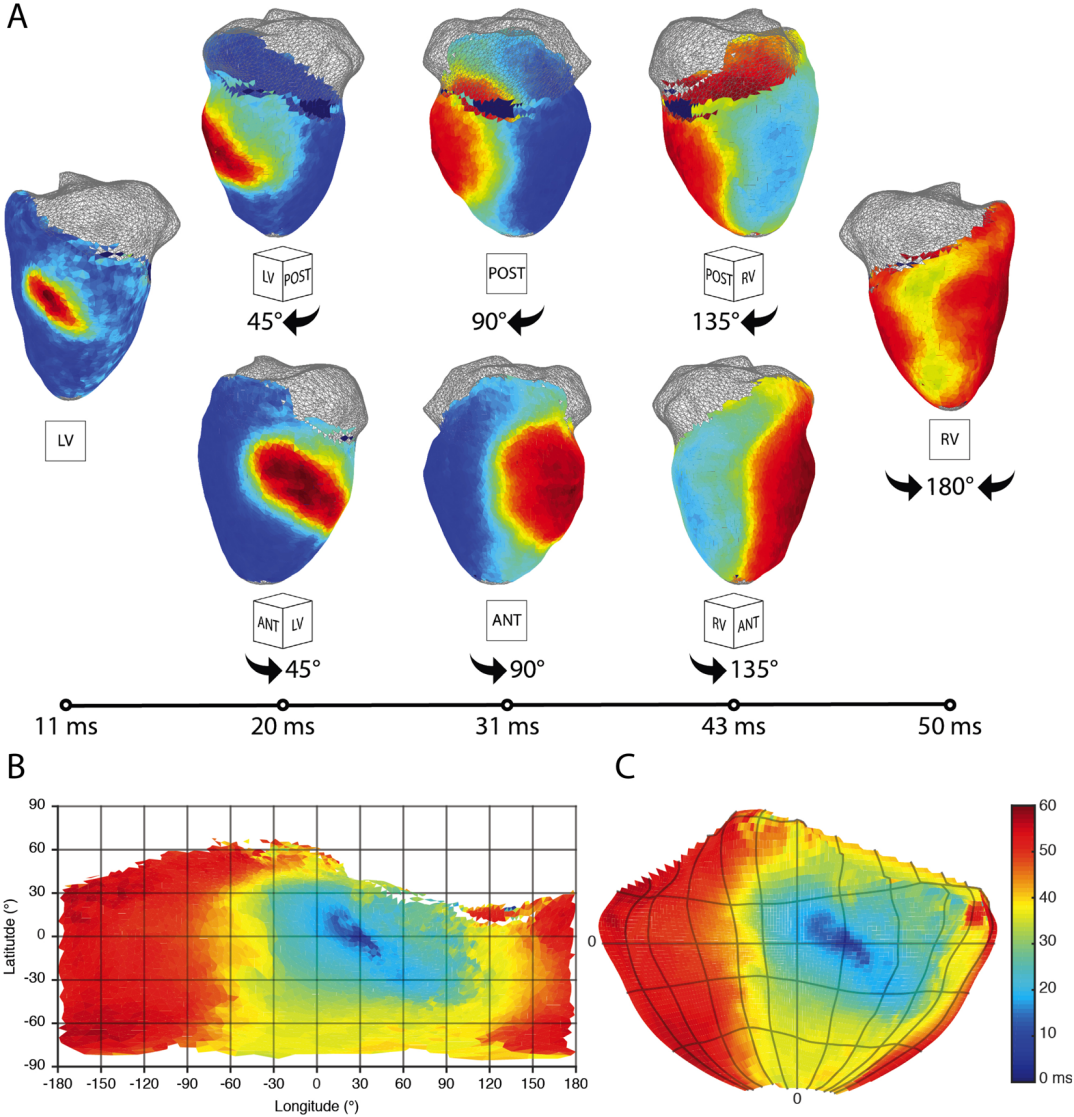
Activation in representative rabbit panoramic optical data. (**A**) The time course of pacing activation is tracked using membrane potential. A paced beat starts on the left ventricular free wall and the divergent wavefronts are tracked over time around both the posterior and anterior sides to where they meet on the right ventricular free wall. Wavefronts are uninterrupted by artifact or discontinuity. (**B**) On the left, a 2D Mercator projection of the isochronal activation map generated using the processing GUI. On the right, a 2D Hammer projection whose latitude and longitude divisions are the same as seen in the Mercator projection.

Figure 5 shows panoramically mapped spread of electrical activity in a rabbit heart. The paced beat originates in the left ventricular free wall and spreads towards the both the anterior and posterior sides to finally collide on the right ventricular free wall (Fig. 5A). The corresponding activation can be visualized using a flat Mercator projection (Fig. 5B) or a Hammer projection (Fig. 5C) in which ratios are maintained.

**Figure 6:**
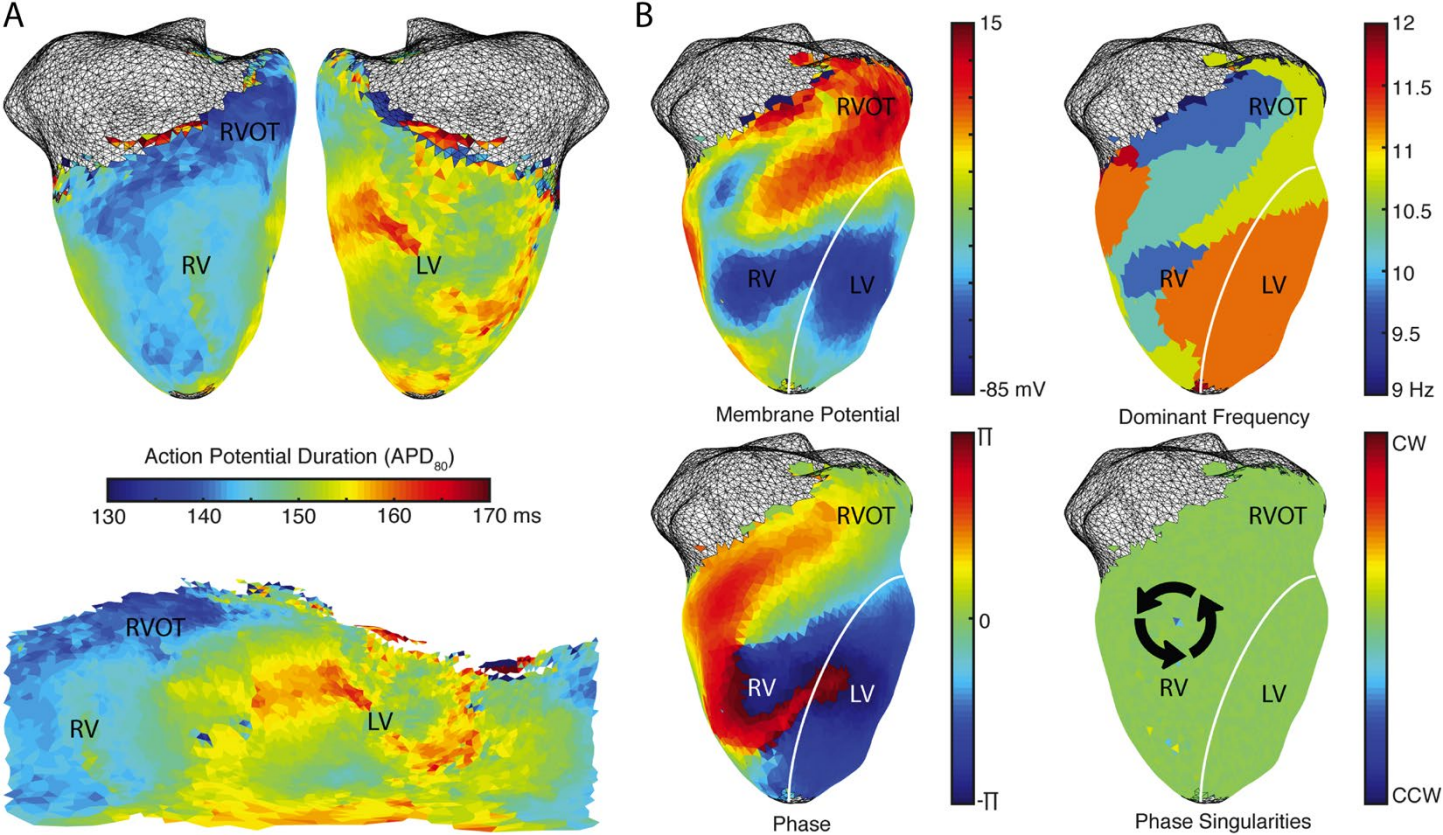
Repolarization and arrhythmia analysis in representative rabbit data. (**A**) Action potential duration can be calculated using projected data and unwrapped into a Mercator 2D representation. (**B**) Arrhythmias like ventricular fibrillation can be analyzed using calculations of dominant frequency, phase, and phase singularities.

Figure 6 shows action potential duration maps (Fig. 6A). Ventricular fibrillation was pharmacologically induced in the heart and mapped for subsequent analysis including dominant frequency calculation as well as generating phase and phase singularities maps (Fig. 6B).

**Figure 7:**
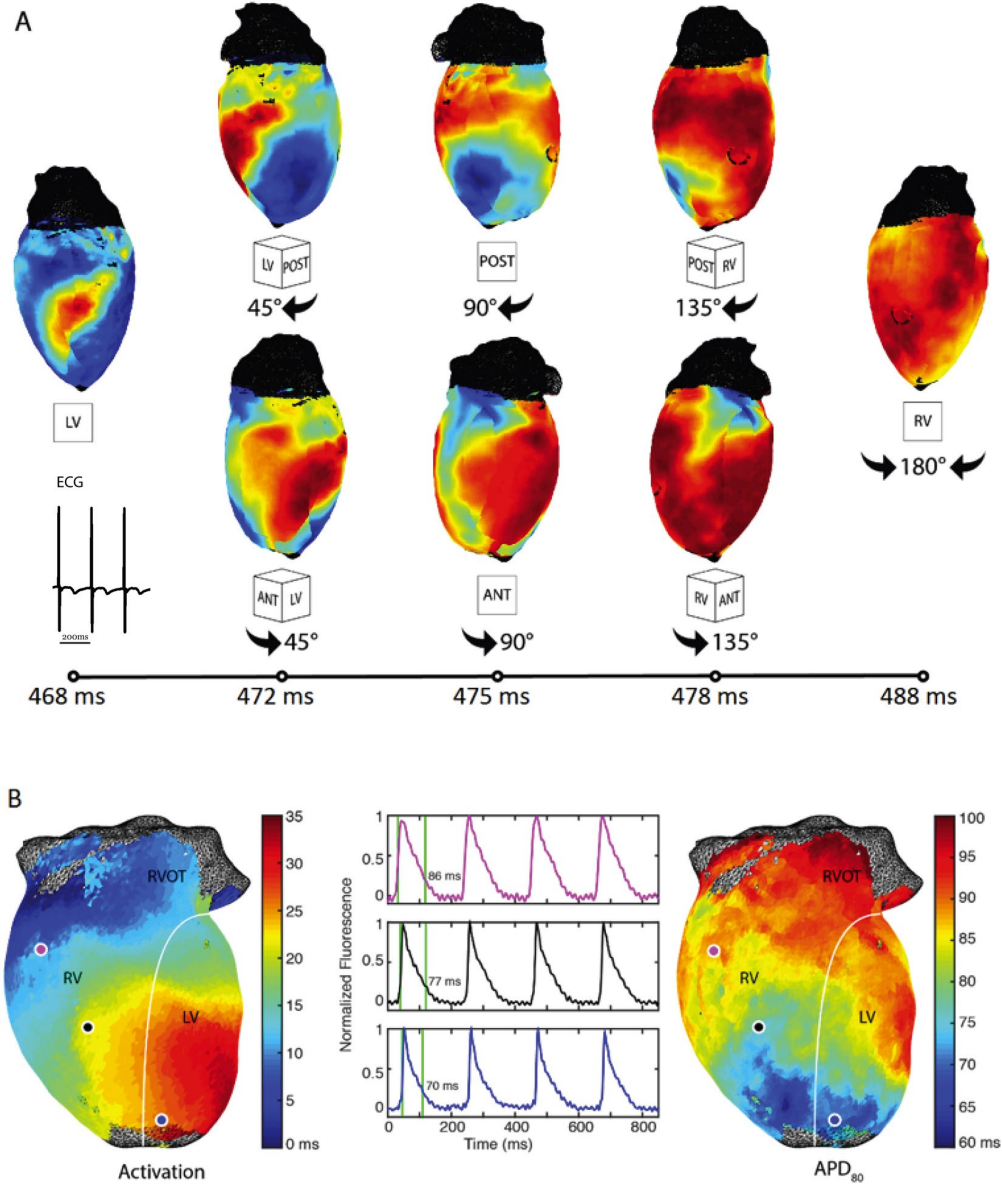
Representative rat panoramic optical data. (**A**) The time course of pacing activation is tracked using membrane potential. (**B**) Activation and the corresponding action potential duration (APD80) maps are seen along with representative action potentials.

Figure 7 depicts the panoramically mapped propagation wave in a rat heart. A paced beat is similarly tracked over time around both the posterior and the anterior sides to where they meet on the right ventricular free wall (Fig. 7A). The activation and action potential duration maps as well as sample optical action potentials are also depicted (Fig. 7B).

**Figure 8:**
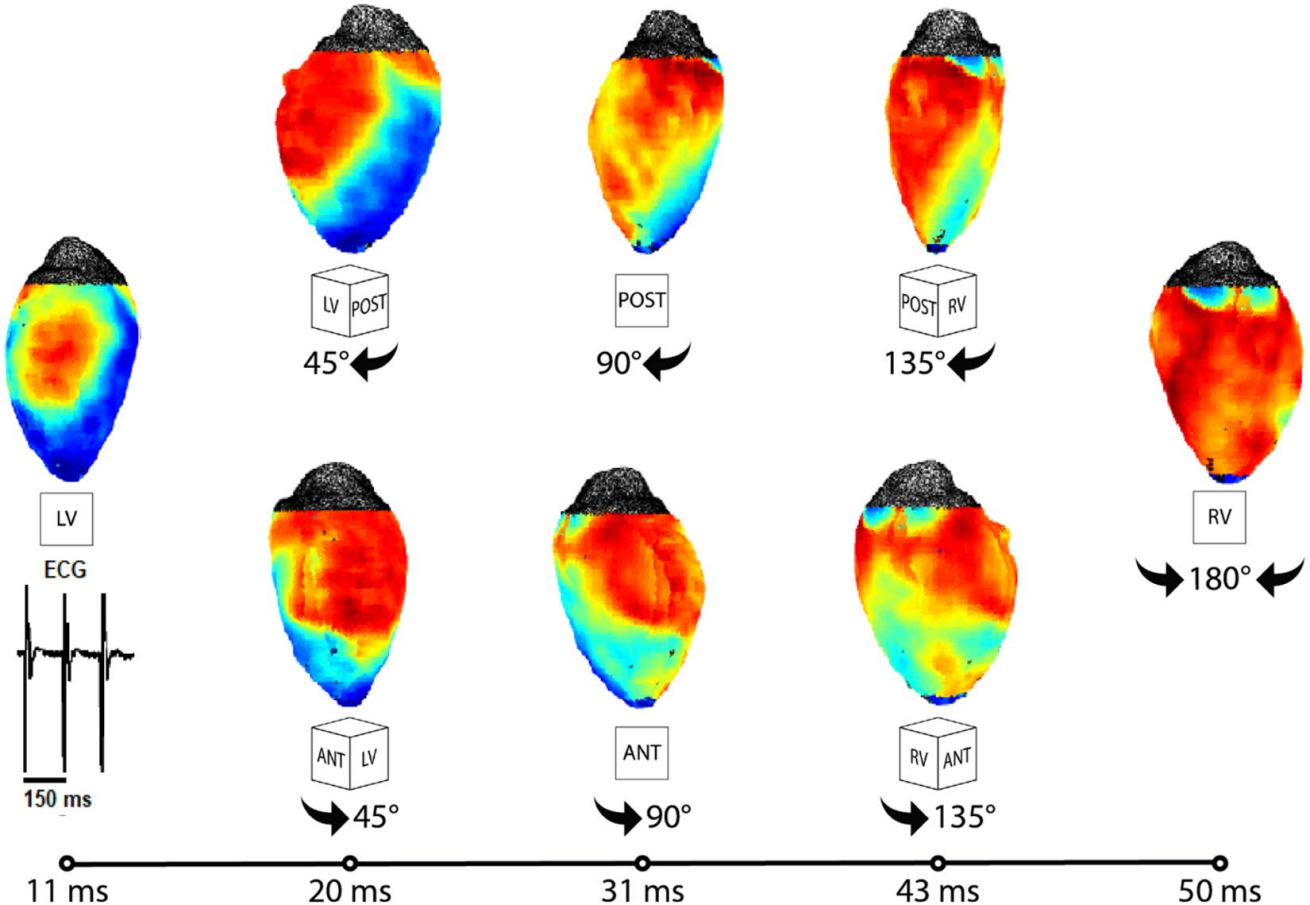
Representative mouse panoramic optical data. (**A**) The time course of pacing activation is tracked using membrane potential.

Figure 8 likewise captures the propagation wave in a panoramically imaged mouse heart.

## Discussion

In this study, we developed an open source panoramic optical imaging system suitable for the whole heart imaging of small mammalian hearts. While panoramic imaging was developed over a decade ago, few studies of actual physiology have been performed, and only by groups that developed panoramic methodologies. We believe this is in large part because this technology has always required significant engineering experience, which has acted as a barrier to entry for many in the cardiac physiology community. There has been a recent effort to present a low-cost panoramic imaging system^33^. While it addresses the issue of cost, the effort still falls short of providing publicly available and curated software for acquisition, processing, and analysis of both anatomical and optical data acquired from the complex 3D surface of the heart. Another contributing factor has been the explosion of transgenic small rodent models for looking at atrial and ventricular arrhythmias, which before now could not be panoramically imaged. Our open source system successfully simplifies the process of data acquisition, processing, and analysis making it more accessible to those without engineering expertise. It also facilitates the imaging of small hearts (e.g., mouse and rat), successfully opening the door to a host of relevant, but incompletely understood, transgenic models.

## Methods

Study subjects included rabbit, rat and mouse, following the approval from the George Washington University’s Animal Care and Use Committee and conforming to the Guide for the Care and Use of Laboratory Animals (NIH Pub. No 85–23, Revised 1996).

### Experimental Setup

**Figure 9:**
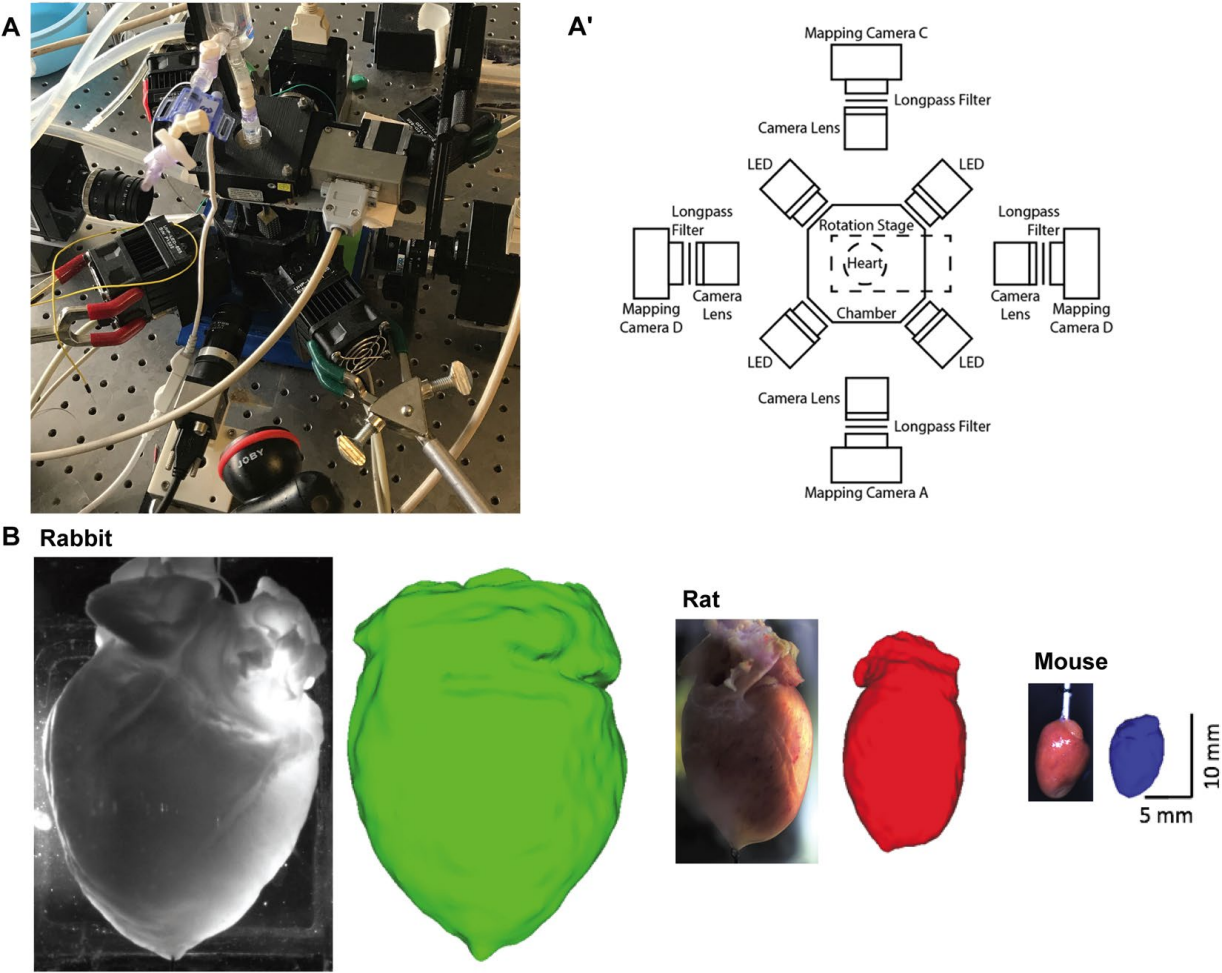
Panoramic optical mapping system. (**A**) Picture and schematic of the optical mapping system are shown. In addition to increasing the number of optical cameras, we designed, and 3D printed a number of experimental components including the perfusion chamber. (**B**) Representative rabbit, rat and mouse hearts and the corresponding reconstructed geometries.

Previous optical mapping systems have been described in detail^3,4,34^. We limit our description to the characteristics unique to this open source panoramic system. Our panoramic imaging setup (Fig. 9A,A’) consists of four CMOS Ultima cameras attached to a MiCAM05 acquisition system (SciMedia Ltd, Costa Mesa, CA). These cameras are mounted to magnetic bases and each outfitted with 35 mm C-mount lens (MLV35M1, Thor Labs, Newton, NJ) and 655 nm long pass filters (ET655lp, Chroma Technology Corporation, Bellow Falls, VT). At 45° angles between the cameras are four 630 nM red LEDs (UHP-T-LED-630, Prizmatix Ltd, Givat-Shmuel, Israel). The perfusion system runs into a cannula mounted to a rotational stage (URS50, Newport Corporation, Irvine, CA). The stage is powered and rotated using a custom Labview interface, Arduino, and stepper driver. A parts list, code, and assembly instructions are included in the Data Supplement.

Recently, multiple labs have published on their efforts to use 3D printing to economically and independently customize their systems to specific experimental needs^35,36^. Here we present several potential applications of 3D printing in customizing a panoramic imaging system. We used a hanging Langendorff-perfused heart in a 3D printed superfusion chamber (Fig. 9A’). The tissue chamber was designed to maximize accessibility to heart for cameras and LEDs, while minimizing the volume to minimize the quantity of drug required to reach desired concentrations. Because we use a superfusion chamber, it is necessary to rotate the heart in front of the camera rather than the camera around the heart, when collecting images for geometric reconstruction. To prevent the perfusion tubing from becoming tangled, we designed, and 3D printed a cannula mount that attaches to the rotating face of the rotational stage directly above the heart and a bubble trap mount to be positioned on the opposite side of the stage. Connecting the bubble trap and the cannula is a female-to-female luer lock connector with an O-ring and swivel that allows the two ends of the connector to spin independent of one another, thus preventing entanglement. Additionally, a platform was designed and printed to facilitate anchoring of the cardiac apex without obscuring the field of view of the cameras or LEDs. Items not shown include camera mounts, positioning brackets, and a backdrop for increasing image contrast during rotation of the heart. Images, design specifications, and files for all components are included in the Data Supplement.

Design of all components was either done in entirely free (e.g. Autodesk Student Version, Autodesk Inc, San Rafael, CA) or well-discounted (e.g. SketchUp Pro Academic License, Trimble Inc, Sunnyvale, CA) CAD software packages. Once completed, the designs were exported to the stereolithography format (i.e. *.stl) and a printer path was created using either the Stratasys Insight 10.8 or Stratasys Control Center 10.8 software packages. Printing was done on both a Stratasys Fortus 250mc and a Stratasys uPrint SE Plus (Stratasys, Eden Prairie, MN) using acrylonitrile butadiene styrene (ABS) plastic. Print time varied from 2 hours for the smallest components to over 30 hours for the large chamber. It should be noted that sophisticated 3D printers can themselves be cost prohibitive, but 3D printing can be outsourced to on line commercial printers at low cost. Similar designs can still be accomplished using entry-level 3D printers, but different design approaches must be used to compensate for their general lack of dissolvable support materials.

The design of this panoramic imaging toolkit, in particular the use of 3D printed components, makes it easily scalable for hearts of varying sizes (Fig. 9B). To demonstrate this, we conducted optical imaging studies in rabbit (left), rat (middle) and mouse (right). Typically, the rat heart (2.1 cm external length, 1.2 cm external width) is half the size of a rabbit heart, whereas the mouse heart (1 cm length, 0.4 cm width) is ¼ the size of a rabbit heart.

### Optical Mapping

The rabbits were initially anesthetized using a ketamine and xylazine cocktail. We administered a dose of heparin and brought them to a deeper level of anesthesia using isoflurane. Once the reflex test failed to illicit a response, we excised the heart using a sternal thoracotomy and immediately cannulated the aorta on a secondary Langendorff perfusion setup. We allowed 3–5 minutes for the beating heart to clear coronary circulation and cardiac cavities from blood before placing it on the panoramic perfusion system and lowering it into the tissue chamber. Once in the chamber, we allowed 10 minutes for the heart to recover from explantation and cannulation procedures. After the recovery period, we added the excitation-contraction uncoupler blebbistatin (Sigma-Aldrich, St. Louis, MO) into the perfusate and brought the perfusate up to a final concentration of 10 μM by slowly injecting blebbistatin into a drug port just upstream from the cannula bubble trap. Once contraction was suppressed, we added the voltage sensitive dye di-4-ANBDQBS (Loew Laboratory, University of Connecticut, Storrs, CT) to the bubble trap up to a final concentration of 35 μM before beginning the experimental protocol.

We first conducted a standard S1S1 pacing restitution protocol at decreasing pacing cycle length (PCL) of 300, 270, 240, 210, 180, and 150 ms. We then administered a 30 μM dose of the ATP channel activator Pinacidil (Sigma-Aldrich, St. Louis, MO) to shorten action potential duration and thus create a substrate that could sustain a reentrant ventricular arrhythmia. After allowing 10 minutes for the drug to take full effect, we induced VF by 1-second burst of 60 Hz pacing. Initialization and maintenance of the arrhythmia were panoramically mapped at multiple time points.

The rat and mouse studies followed similar Langendorff perfusion setup and pacing restitution protocol as described for the rabbit study.

## Electronic supplementary material

Supplementary Information
