# Supplementary material for: RHYTHM: An Open Source Imaging Toolkit for Cardiac Panoramic Optical Mapping
Source: Sci Rep. 2018 Feb 13;8:2921. doi: 10.1038/s41598-018-21333-w (PMC5811559; doi:10.1038/s41598-018-21333-w)
Supplement: Supplementary file 1 — Supplementary Information [file 41598_2018_21333_MOESM1_ESM.pdf]

RHYTHM: An Open Source Imaging Toolkit for Cardiac Panoramic Optical Mapping

Christopher Gloschat[1], Kedar Aras[1], Shubham Gupta[1], N. Rokhaya Faye[1], Hanyu Zhang[2], Roman A. Syunyaev[3], Roman A. Pryamonosov[3,4], Jack Rogers[2], Matthew W. Kay[1], and Igor R. Efimov[1,3*]

[1]The George Washington University, Department of Biomedical Engineering, Washington, 20052, USA
[2]The University of Alabama at Birmingham, Department of Biomedical Engineering Birmingham, 35294,
USA
[3]Moscow Institute of Physics and Technology, Dolgoprudny, 141701, Russia
[4]Institute of Numerical Mathematics of the Russian Academy of Sciences, Moscow, 119991, Russia
[*]efimov@gwu.edu

Supplementary Figures

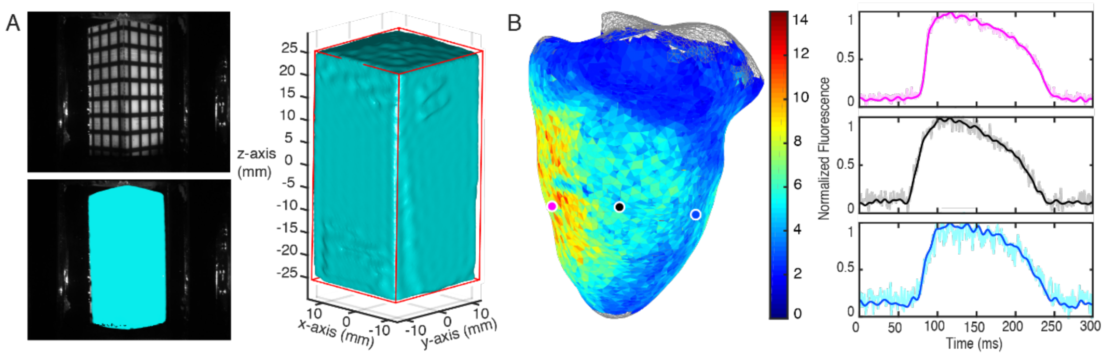

Supplementary Figure 1. Geometric accuracy and signal-to-noise ratio (SNR). (A) To validate the geometric accuracy, an object of known volume and geometry (i.e. the calibration cuboid) was reconstructed. The reconstructed volume was within 3% of the measured volume of the cuboid. **(B)** Subtle inhomogeneities in lighting result in measureable differences in signal quality. Uniform lighting, not only benefits SNR, but can improve camera calibration.

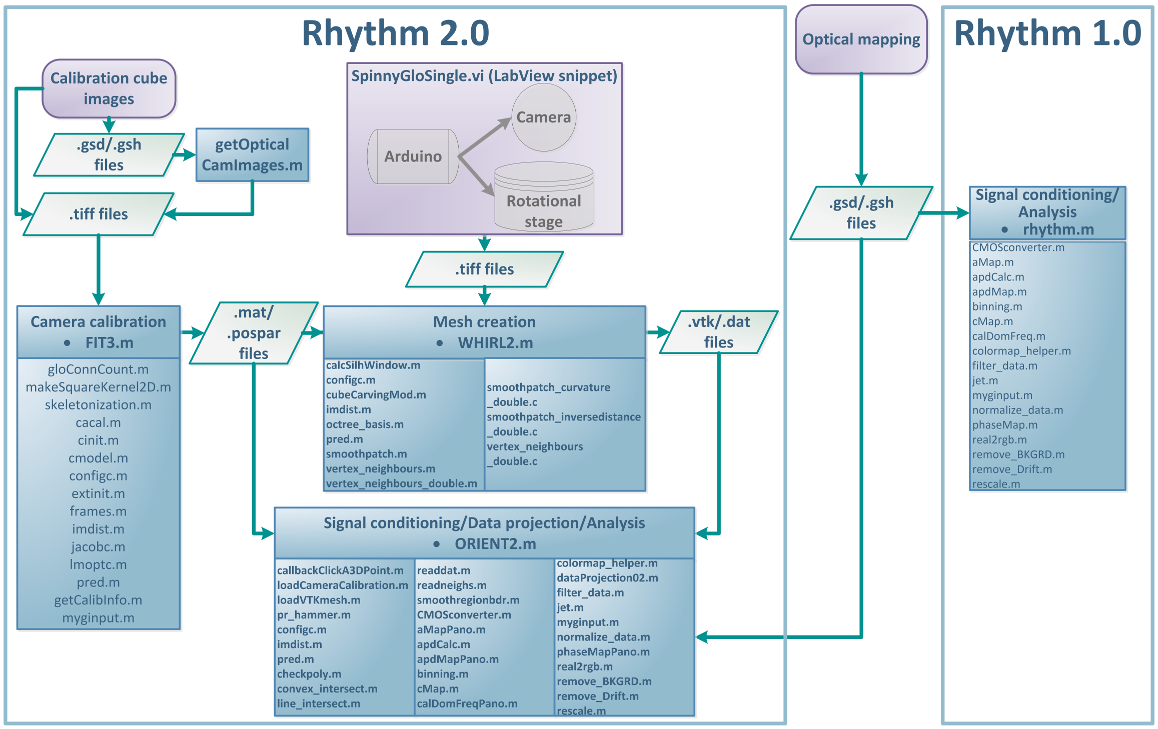

Supplementary Figure 2. Software Architecture. General scheme of software structure and data file format. Rhythm 1.0 (right-hand side) is intended for single camera users. Rhythm 2.0 (left-hand side) is intended for panoramic mapping.

Supplementary Files

The software for acquiring, processing, and analyzing the resultant panoramic has been uploaded to GitHub at https://github.com/optocardiography. Among the files is a README.txt file which provides instructions for installation and documentation which provides an explanation of use. At the same URL are located a document outlining the 3D printed components and the files required to print them.
